# Notes on a Case of Aphonia, Treated by the Method of Oliver*Amr. Jour. Med. Science, April, 1870.

**Published:** 1875-11

**Authors:** W. W. Potter

**Affiliations:** Mt. Morris, N. Y.


ART. II. *Notes on a Case of Aphonia due to Paralysis of Intrinsic Muscles of the Larynx, and its Treatment by its External Manipulation after the Method of Oliver.** By W. W. POTTER, M. D., of Mt. Morris, N. Y.

Mrs. ——, æt. 41, married 17 years—mother of three children—highly neurotic temperament—has lost her voice at periods since she was 20 years of age, and at one time for ten months prior to her marriage. She could not speak above a whisper.

December 25, 1873, she again lost her voice and did not regain it until May 16, 1874—5 months nearly. During this time galvanism, chalybeates, counter-irritation, strychnine, inhalation, etc., were used persistently but all to no purpose so far as the Aphonia was concerned, although the patient's health was very much improved thereby. Failing with these means, as a *dernier ressort*, having by laryngoscopic examination ascertained that there was partial or incomplete paralysis of the vocal cords, I determined to try the method of Oliver, which, though novel, was so completely successful that I shall ask to trespass upon your time with a narrative of the details of its employment:

On the 14th, of May, 1874, I placed the patient in a chair, sitting erect, and with the thumb and forefinger of my right hand compressed the rings of the thyroid cartilage gently but firmly, and directed her to make an attempt to speak. In the course of a few moments a coarse guttural sound was produced, but as soon as the compression was removed the ordinary whisper reappeared. The compression was kept up with occasional moments of rest for

*Amr. Jour. Med. Science, April, 187[illegible].

15 or 20 minutes, but with no other results than the guttural sound before referred to. Not wishing to fatigue my patient, I desisted from further attempts for the day, and left with an appointment for another seance at 11 o'clock next morning. The pressure was applied in like manner at this time and the patient directed to pronounce the *vowels* a, e, i, o, during forced inspiration. A feeble voice sound was soon produced, but nothing more. Next day another trial was made, and this time the sounds were clearer and more natural than before, but the whisper returned soon after the compression was removed. I directed her to practice the attempt to read aloud during the afternoon, and about 5 o'clock she sent me word that her voice had returned. It was some days, however, before it resumed its natural sound. This was on the 16th of May, and between that time aud October 20th, the voice was lost several times at intervals varying from one to four weeks, but was always restored at a single sitting, and has not been lost since the latter date, viz, October 20.

I shall not at this time enter into a discussion of the pathology of Aphonia. My object being simply to call attention to this method of treatment in these cases which was first brought to the notice of the profession by Dr. Henry K. Oliver, physician to the Massachussetts General Hospital, Boston, in a paper published in the Amr. Jour. Med. Science for April, 1870, and in which he reports some half dozen cases successfully treated in this manner.

